# Enhanced DSSCs efficiency via Cooperate co-absorbance (CdS QDs) and plasmonic core-shell nanoparticle (Ag@PVP)

**DOI:** 10.1038/srep25227

**Published:** 2016-05-04

**Authors:** Omid Amiri, Masoud Salavati-Niasari, Samira Bagheri, Amin Termeh Yousefi

**Affiliations:** 1 Institute of Nano Science and Nano Technology, University of Kashan, Kashan, P.O. Box 87317-51167; 2 Nanotechnology & Catalysis Research Centre (NANOCAT), IPS Building, University of Malaya, 50603 Kuala Lumpur, Malaysia; 3 Graduate School of Life Science and Systems Engineering, Department of Human Intelligence Systems, 2-4 Hibikino, Wakamatsu, Kitakyushu 808-0196, Japan

## Abstract

This paper describes cooperate the co-absorbance (CdS QDs) and the plasmonic core-shell nanoparticles (Ag@PVP) of dye synthesized solar cells in which CdS QDs and Ag@PVP are incorporated into the TiO_2_ layer. Cooperative nanoparticles show superior behavior on enhancing light absorption in comparison with reference cells. Cooperated DSSC exhibits the best performance with the power conversion efficiency of 7.64% which is superior to that of the free–modified DSSC with the PCE of 5%. Detailed studies offer an effective approach to enhance the efficiency of dye synthesized solar cells.

The dye-sensitized solar cells (DSSCs) have been regarded as a promising branch of study contrasted to conventional silicon-based photovoltaic devices for sustainable energy supply at low cost and high environmental friendliness^1^. Titanium dioxide (TiO_2_) which absorbs the sunlight in the ultraviolet region^2^ is one of the most prominent oxide materials for performing various kinds of industrial applications such as photovoltaic^3^, photocatalytic^4^,^5^. The wide band gap of TiO_2_ limits its absorption to the ultraviolet region of the solar spectrum^6^. In addition, the low electron mobility of TiO_2_ leads to inferior conversion efficiency of solar cells^7^,^8^. Until the present time, many assays have been done by focusing on the development of high performance sensitizers (e.g. different dyes) to enhance light harvesting in the visible light region^9^,^10^,^11^,^12^. While the development of new dyes has led to continued improvements in the efficiency of DSSCs in recent years^13^,^14^, they are still restricted by the weak absorption of the dye sensitizer like ruthenium(II) polypyridyl dyes (e.g. N719). N719 absorbs strongly at 535 nm, but it has drastically reduced extinction coefficients at longer wavelengths^15^. Therefore, enhancing the light harvesting efficiency (LHE) in the 300–900 nm wavelength range is considered as a favorable approach to increase the power conversion efficiency (PCE) and photocurrent of these devices^16^,^17^. It is well known that an effective method for trapping light or enhancing light harvesting to develop new photovoltaic devices is utilizing of some noble metallic nanostructures with high scattering cross section^18^,^19^. It has been reported that metal nanoparticles (NPs) such as gold (Au) and silver (Ag) can enhance the photo response of photovoltaic devices by acting as light trapping agents^20^,^21^, photosensitizers^22^, and electron traps for facilitating charge separation^23^,^24^. These nanoparticles have a strong optical behavior in the visible region due to surface plasmon resonance (SPR) effect due to collective electron oscillation^25^.

Also Quantum dot-sensitized solar cells are considered as a promising candidate for the progression of next generation solar cells due to their simple and low cost fabrication techniques. Some quantum dots (QDs) such as CdS, CdSe, CdTe, PbS and so on, which absorb light in the visible range, can serve as co-sensitizers so after absorption of a photon with enough energy, they are able to transfer electrons to the conduction band of TiO_2_^6^. Among these QDs, CdS with suitable band gap and band positions compared to the conduction band of TiO_2_ can create a long distance charge separated states with electron and hole at sites far from each other, so it’s proper for using in these devices, in order to improve energy conversion efficiency of QDSSCs^13^,^14^. However, the relatively low conversion efficiency of such cells is still a primary challenge for the large-scale applications of QDSSCs. QDs have the advantage of a broad absorption spectra compared to molecular dyes with narrow absorption spectra. Moreover, photochemical reactions, particularly with the liquid electrolyte can induce significant degradation of the QD sensitizers. One can find detailed accounts related to these subjects in two recent reviews^26^,^27^.

In this work we have reported a cooperative PCE enhancement of 60% by adding CdS QDs and Ag@PVP nanoparticles mixture into TiO_2_ layer.

We have presented a design which combines the benefits of QDs in terms of their broad absorption spectrum and the light harvesting efficiency (LHE) of noble metal (Ag@PVP). In this design, QDs show significant absorption between 200–300 nm, while N719 dye molecules show absorption in 313 nm and 500–560 nm, and Ag@PVP NPs have absorption in 350–450 nm (Fig. 1).

In addition, the effects of different weight volume percent (w/v) and treatment time of Ag@PVP nanoparticles (NPs) on performance of devices were studied.

## Result and Discussion

**Figure 2:**
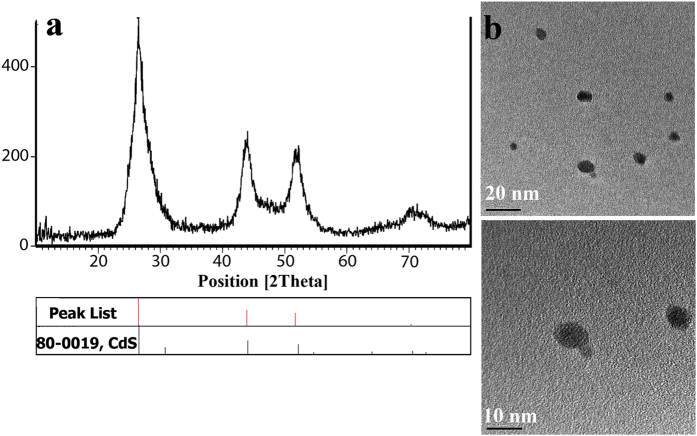
(**a**) XRD pattern of as synthesized CdS QDs, (**b**) related HRTEM.

Here, we have introduced a very simple and low cost method for synthesis of CdS QDs. In this method, CdS QDs were prepared by co-precipitation method in which mixture of water, propylene glycol, and ethanol were used as solvent in 40–50 °C. Figure 2a,b show the XRD pattern and HRTEM of as-synthesized CdS quantum dots where CdS QDs were prepared with no impurity and uniform size.

**Figure 3:**
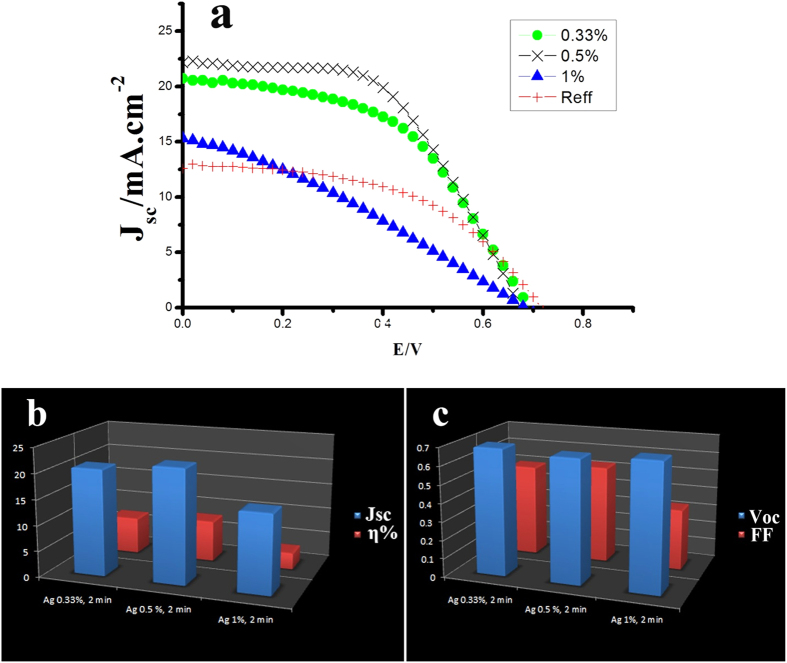
(**a**) J-V curve of different devices including different weight percent of Ag@PVP (**b**) JSc & ◻% -weight percent of Ag@PVP and (**c**) Voc & FF -weight percent of Ag@PVP.

In order to investigate the effect of CdS QDs and Ag@PVP NPs in DSSCs, we first optimized device performance based on Ag@PVP NPs, and then we investigate their cooperative effect on DSCs performance a case by case. The devices were assembled with different weight percent of Ag@PVP from 0.33% to 1%. These devices were fabricated and measured under AM 1.5 illuminations at 100 mW/cm^2^. Corresponding current density versus voltage (J–V) curve and details of DSCs based on the different weight percent of Ag@PVP NPs are reported in Fig. 3 and Table 1.

The best efficiency of cells which immersed in 0.33% of Ag@PVP for 2 min was 7.14% (average efficiency was 7.04%). It shows 43% improvement compared to reference cells (5%). 8.01% efficiency (average 7.9) was achieved by increasing Ag@PVP concentration into 0.5% for 2 min. When photoanodes immersed in 1% of Ag@PVP, efficiency decreased to 3.2%. At first, by increasing the concentration of plasmonic materials to 0.5%, efficiency increased sufficiently because more N719 molecules are effected by near field of plasmonic materials while high concentrations of Ag@PVP can act as trap center and decrease the efficiency. Therefore, efficiency decreased to 3.2 (average 3) in the present of 1% Ag@PVP.

According to our previous work, we found that treatment time has a sufficient effect on the efficiency^28^. Thus, we optimized the treatment time for immersing photoanode in Ag@PVP solution. We investigated three treatment time (1, 2, and 3 min). As seen in Fig. 3, optimum treatment time is 2 min. Treatment time has the same effect as concentration of Ag@PVP. The efficiency of the devices with 1 min treatment was 6% (average 5.9%) which shows better performance compared to reference cells and efficiency of cells with 2 min treatment was 7.14%.

**Figure 4:**
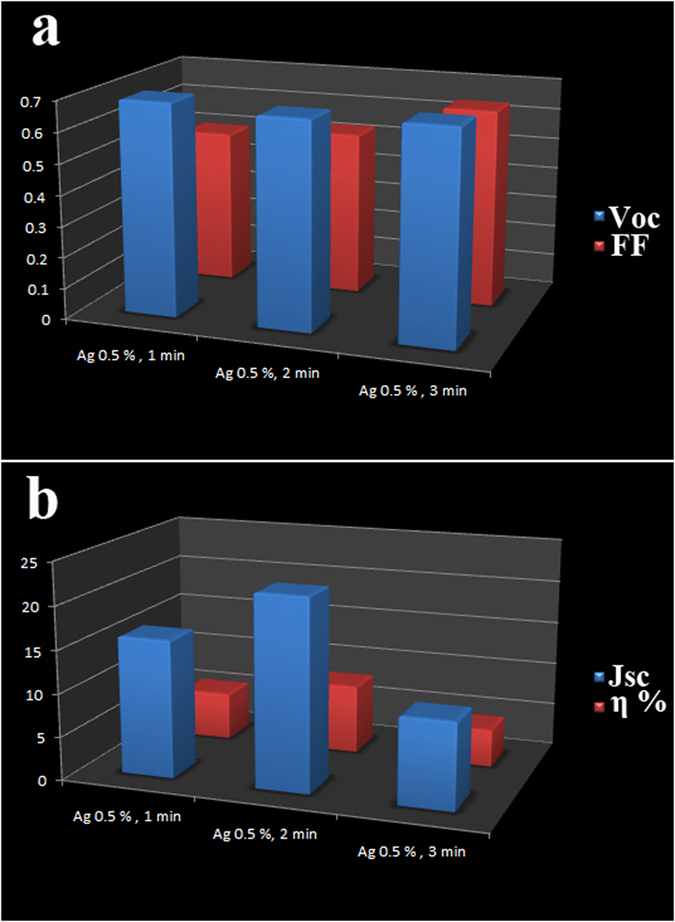
(**a**) Voc & FF –time treatment of Ag@PVP and (**c**) JSc & η% - time treatment of Ag@PVP.

While increasing time treatment to 3 min showed the reverse effect on performance of DSSCs (Fig. 4 and Table 1).

After that, we studied the cooperation effect of CdS QDs with Ag@PVP NPs by simply mixing them in optimized conditions together into TiO_2_ layer. Very interestingly, devices with CdS QDs/Ag NPs had much better solar cell performance.

**Figure 5:**
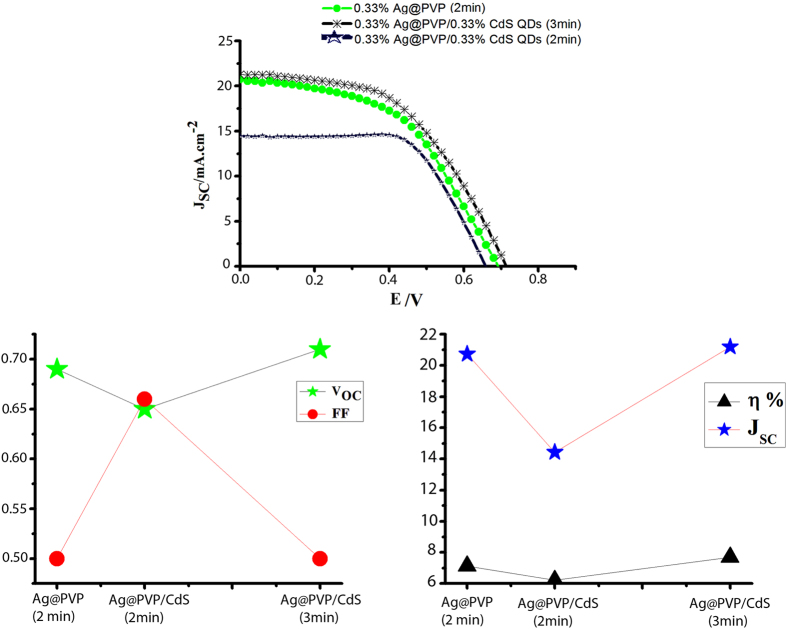
(**a**) J-V curve of different devices including Ag@PVP/CdS QDs (**b**) JSc & η% -time treatment of Ag@PVP/CdS QDs and (**c**) Voc & FF–time treatment of Ag@PVP/CdS QDs.

We chose 0.33% w/v of Ag%PVP and CdS QDs to investigate their cooperation. Then we immersed the photoanodes in this solution for 2 min. Typically, solar cells with CdS QDs/Ag@PVP NPs showed 6.21% efficiency (average 6.19%) which is lower than 7.14% for solar cells with Ag@PVP NPs. So we increase treatment time to 3 min led to improve efficiency to 7.65% which shows 7% improvement compared to 0.33% of Ag@PVP (Fig. 5 and Table 1). As shown in Fig. 5 and Table 1, Jsc was reached to 22.5 by cooperation of Ag@PVP and CdS QDs.

**Figure 6:**
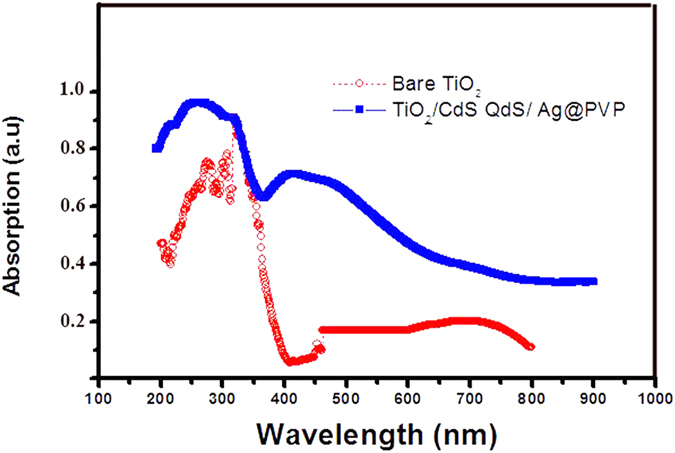
DRS spectra of reference cell and cell including CdS QDs/Ag/PVP.

Figure 6 shows the DRS spectra of reference cell and cell including CdS QDs/Ag/PVP. According to this Figure, cell including CdS QDs/Ag/PVP shows broad absorption from 200–600 nm, which peak in 350–450 nm refers to the plasmonic peak of Ag@PVP.

The small standard deviations indicate good reliability and reproducibility of our results. Intensity modulated photocurrent spectroscopy IMPS^29^,^30^ and intensity modulated photovoltage spectroscopy IMVS^8^ can be used to measure the respective time constants for the combined processes of charge collection and charge recombination at short circuit and charge recombination at open circuit condition in dye-sensitized solar cells.

It is expected that the loss of injected electrons via both photocurrent from external load and recombination are proportional to the electron concentration in TiO_2_ and the rate constants for these processes are independent of each other^31^. The time constant for charge recombination at open circuit (τr) was obtained from IMVS. The collection (transport) time (τc) can be estimated from the equation τr = 1/2πfc, where fc is the characteristic frequency maximum of the IMPS imaginary component and the charge-collection efficiency ηcc is given by the equation ηcc = 1−(τc/τr). IMPS and IMVS experiments were done by a modulated AC light from 1 to 1000 Hz superimposed on a relatively large bias illumination at 470 nm.

**Figure 7:**
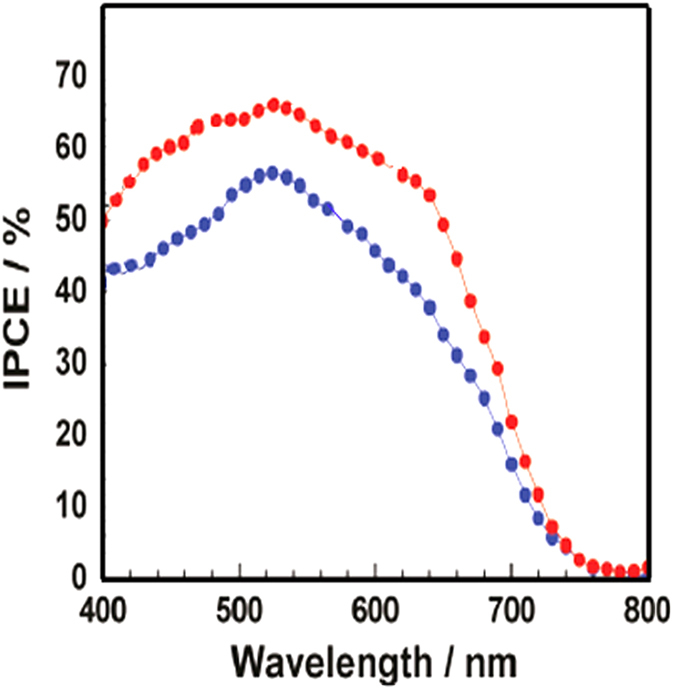
IPCE spectra of control device with best efficiency and devices including CdS QDs and Ag@PVP.

The effect of CdS QDs, and Ag@PVP NPs on transport, recombination times, and charge-collection efficiency are presented in Table 2. It is clear that devices based on CdS QDs and Ag@PVP NPs show higher collection time and charge-collection efficiency. IPCE was used for explaining the improved PCEs. Figure 7 shows the IPCE spectra of control device with best efficiency and devices including CdS QDs and Ag@PVP. It is indicated that the IPCE of devices including CdS QDs and Ag@PVP is increased over the whole wavelength range, especially within 400–700 nm, compared with that of TiO2/N719-only DSCs. The IPCE is related with the light-harvesting efficiency, electron injection efficiency, and carrier collection efficiency simultaneously. Assuming the electron injection and collection are seldom affected, the increase of the IPCE should be attributed to the light harvest enhancement due to the CdS QDs and Ag@PVP.

**Figure 8:**
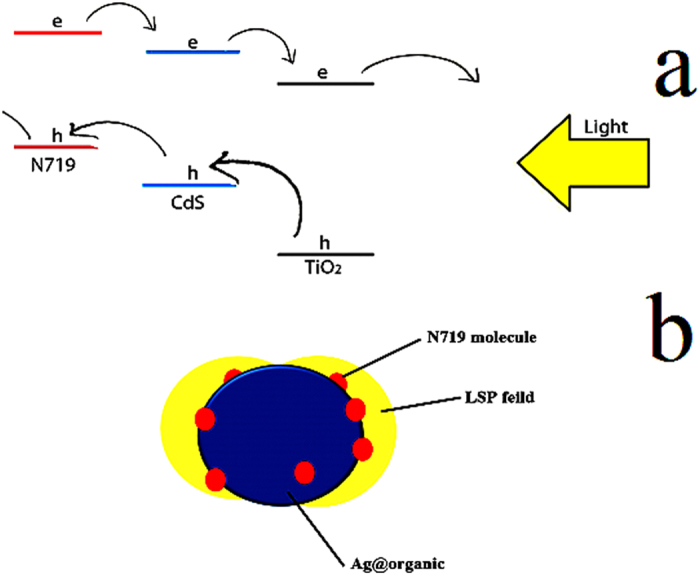
Detailed mechanism for enhancing efficiency in CdS QdS/Ag@PVP cell.

Base of these results, we illustrate a mechanism for this improvement (Fig. 8). As shown in mechanism, PCE could improve in 2 ways. First, CdS QDs can act as co-sensitizer. It means CdS absorb certain wavelengths and produce electrons and holes which can transfer to the external circuit. Also conduction band of CdS is located between the conduction band of dye and TiO_2_. Therefore electrons can jump from the conduction band of dye to the conduction band of CdS and finally to the conduction band of TiO_2_ before there are recombined with holes in valance band of dye (Fig. 8a). Second, the local plasmonic resonances arising from Ag@PVP core shell nanoparticles increase the optical absorption of dye molecules in DSCs. PVP has the advantages of not only readily forming films, but also can chemically and electronically protect the silver from the corrosion and recombination. Besides, PVP have the unique ability of adhesion to dye molecules, which help to trap mass of dye molecules surrounding the silver nanoparticles surface, hence the LSP field from silver sphere can affect sufficient dye molecules, as shown in Fig. 8b, resulting in a significant enhancement on the optical absorption and overall efficiency of the DSCs.

**Figure 9:**
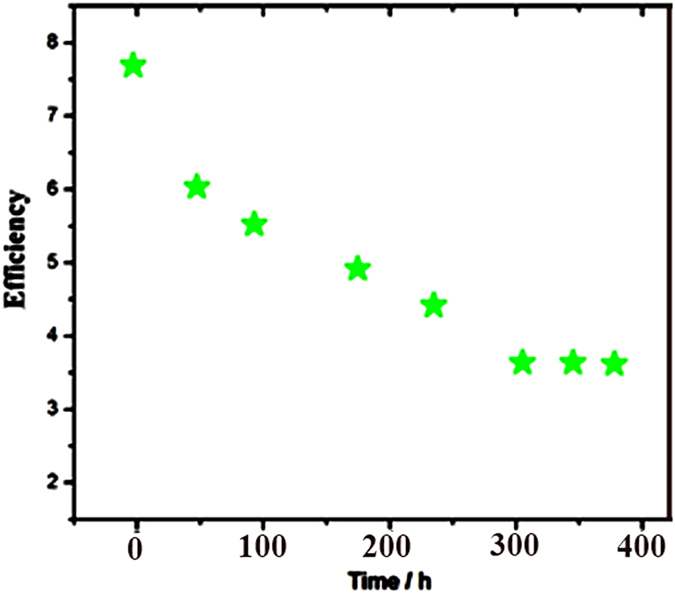
Stability testing of devices including CdS QDs and Ag@PVP under the A.M 1.5.

Figure 9 shows detailed evaluation of the device parameters during the 400 hours measured at room temperature under A.M 1.5. During this time period, PCE decreased 53%. During the first 50 hours PCE decreased suddenly because iodide/triiodide electrolyte corrodes CdS QDs. After 50 hours, this rate (rate of decrease in PCE) decreased and it was stopped after 300 hours.

**Figure 10:**
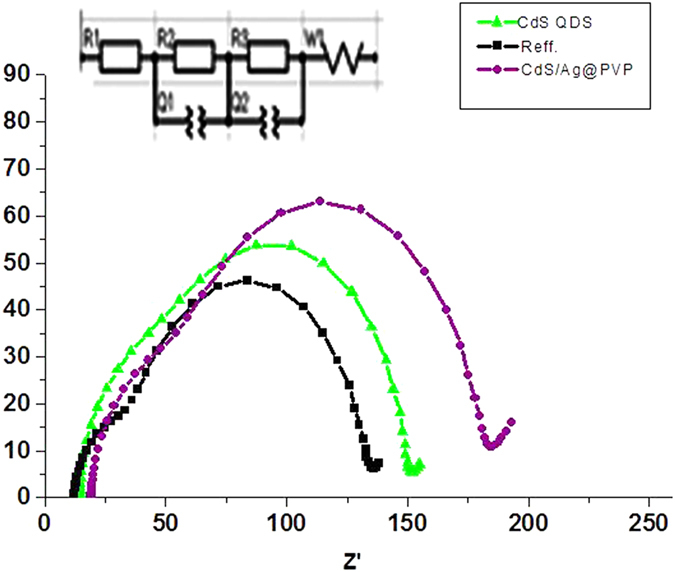
Electrochemical impedance spectra for reference cell and devices containing: CdS QDs and CdS QDs/Ag@PVP.

Figure 10 and Table 3 demonstrate typical Nyquist plots of the DSSCs with bare TiO_2_ and cells containing CdS QDs/Ag@PVP. The impedance spectra were fitted to a simplified version of the equivalent circuit (Fig. 10). The some important parameters of the working electrodes in these devices were determined from the second semicircles in Fig. 10 that the results have been summarized in Table 3. The effective diffusion coefficient of electrons (Deff) can be determined by Eq. 1:


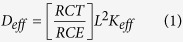


Here, keff is the reaction rate constant for the electron recombination with triiodide and L is film thickness. keff is obtained by the electron lifetime (τ) and angular frequency (ωmax) at the second semicircles in the Nyquist plots of the dye synthesized solar cells (Eqs (2) and (3):


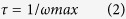



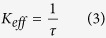


There is small difference between these devices for the RCE at the counter electrode/electrolyte interface. These results show that the CdS QDs/Ag@PVP core shell NPs do not make the over potential at the counter electrode. Notably, the fitted value of R_CT_ for cells containing CdS QDs/Ag@PVP NPs are found ~110 Ω cm^2^, while the corresponding value for reference cells are ~73 Ω cm^2^.

This significant increase in R_CT_ shows that the organic shells are more favorable to suppress the charge recombination process that arises from electrons in TiO_2_ film with triiodide in electrolyte solution and leads to increase Jsc. On the other hand, the adsorption of CdS QDs/Ag@PVP NP on the TiO_2_ surface causes to increase in electron density in the conduction band of TiO_2_. In the cell based on CdS QDs/Ag@PVP NP electrode, the middle-frequency peak in the Nyquist plot shifts to lower frequency relative to reference device, exhibiting a longer electron lifetime for these cells.

## Conclusion

To conclude, we improved the PCEs and charge-collection efficiency of DSCs by incorporating QDs and Ag@PVP NPs into TiO_2_ layer. LSPR induced near field enhancement not only leads to heightened light absorption of active layer materials of DSCs but also benefits charge separation and transport, resulting in increased charge carrier density. Moreover, we also showed that cooperative plasmonic enhancement could be achieved by simply combining CdS QDs and Ag@PVP NPs into TiO_2_ layer. The LSPR and QDs absorption lead to enhance light absorption region after combination; this idea is further proved by solar cell performance of devices with separate CdS, Ag@PVP NPs. We believe that the results of our study offer an effective approach to enhance the efficiency of dye synthesized solar cells.

## Method

### Synthesis of CdS QDs

CdS QDs were prepared according to our previous work^32^. Briefly, CdS QDs were prepared by the reaction of cadmium oxalate with thioacetamide (TAA) in mixture of DI water, ethanol and propylene glycol (PG) as solvent under magnetic stirring at 45 °C. Here, CdS QDs were prepared with a very simple method at 45 °C.

To fabricate Ag@PVP core-shell NPs, monodisperse Ag@PVP NPs are prepared according to the method described in our previous work^33^. Briefly, an aqueous solution containing AgNO_3_ (purchased from Merck) is heated to its boiling point under stirring, and then a hydrazine solution is injected quickly into the system. An aqueous solution of PVP (purchased from Merck) is added to the colloidal Ag solution to modify the Ag NPs. The solution is stirred for 12–18 h at room temperature. The PVP-coating Ag NPs are collected by centrifugation and re-dispersed in ethanol by bath sonication.

### Preparation of working electrode

**Figure 11:**
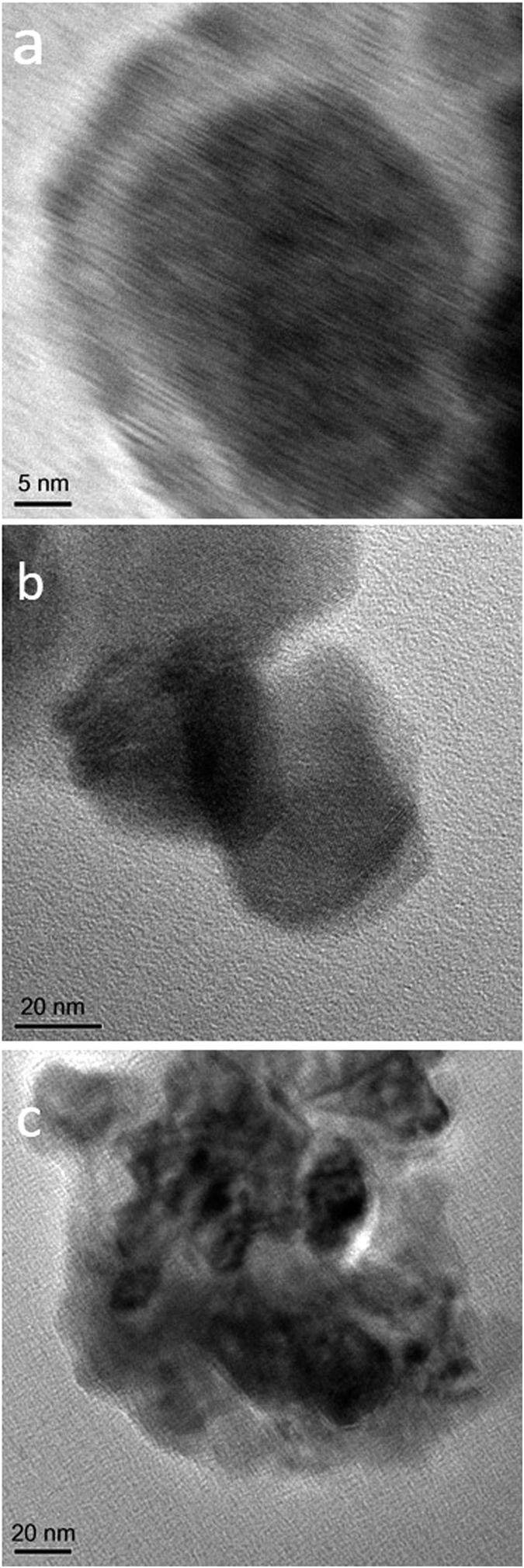
HRTEM of (**a**) as-synthesized Ag@PVP, (**b**) pure TiO2 film and (**c**) TiO2 film, including Ag@PVP and CdS quantum dots.

Electrophoretic deposition (EPD) method was utilized to prepare P25 NPs-based films used in DSSCs. During Electrophoretic deposition, the cleaned FTO glass was remained at a positive potential, anode, while a pure steel mesh was used as the cathode electrode. Power was supplied by a Megatek Pro-grammable DC Power Supply (MP-3005D). During the process the applied voltage was 10 V and the deposition cycles were 4 with each cycle of 15s. The coated substrates were dried at 150 °C. The resulted layer was annealed under air flow at 500 °C for 60 min. Electrolyte solution and the amount of additives are important for creation of a high quality surface as mentioned in other experiment reported previously^32^,^33^. We used optimal concentrations of additives in the electrolyte solution as follows: I_2_ 6 mg, acetone 0.4 ml, and water 1 ml. The electrodes, which were prepared with EPD, were immersed in colloidal solution of Ag@PVP NPs and CdS QDs for 2 min before drying at 100 °C. Figure 11 shows the HRTEM of as-synthesized Ag@PVP (Fig.11a), working electrode before (Fig.11b) and after immersing in colloidal solution of Ag@PVP NPs and CdS QDs (Fig. 11c). As seen from Fig. 11a, size of Ag core and PVP shell are about 20 nm and 5 nm, respectively. Ag@PVP is almost spherical. Figure 11b shows the pure P25 particles with size about 21 nm. HTRTEM of Ag@PVP NPs, CdS QDs and P25 blend is shown in Fig. 11c. As seen in the figure, Ag@PVP NPs and CdS QDs are well dispersed in P25 particles.

## Additional Information

**How to cite this article**: Amiri, O. *et al.* Enhanced DSSCs efficiency via Cooperate co-absorbance (CdS QDs) and plasmonic core-shell nanoparticle (Ag@PVP). *Sci. Rep.* **6**, 25227; doi: 10.1038/srep25227 (2016).

## Figures and Tables

**Figure 1:**
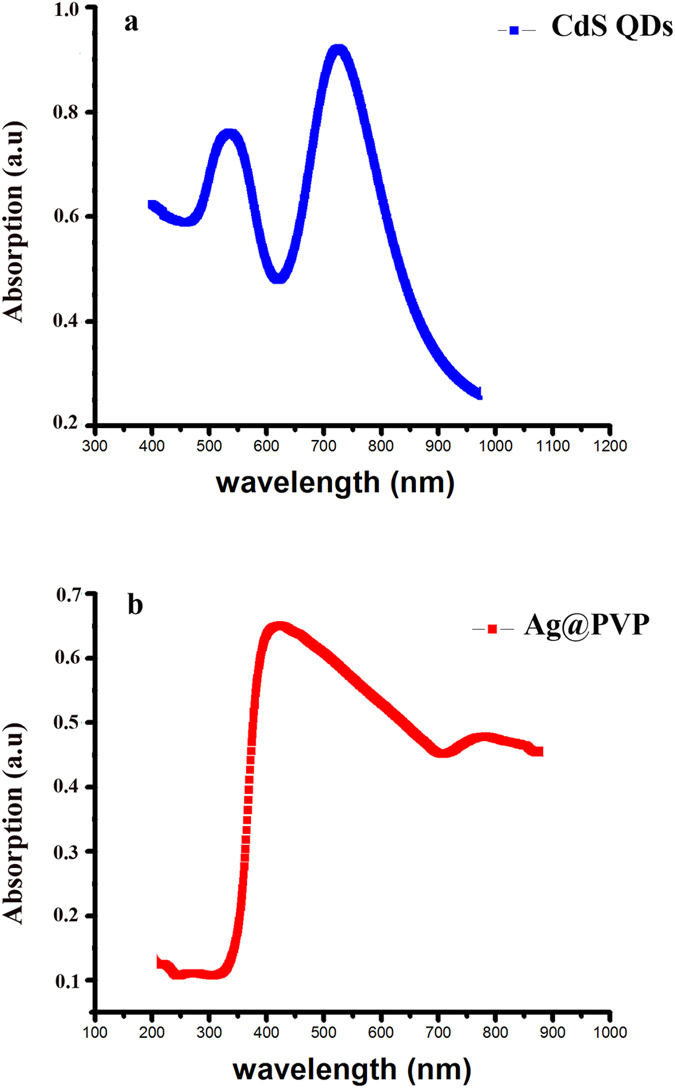
(**a**) Normalized uv-vis spectrum of CdS QDs and (**b**) Ag@PVP.

**Table 1:** Solar cell performance parameters of fabricated devices under AM 1.5.

DSCs	Voc	Jsc	FF	η%
0.33% Ag@PVP	0.69 ± 0.01	20.73 ± 0.15	0.50 ± 0.02	7.14
0.5% Ag@PVP	0.67 ± 0.01	22.16 ± 0.20	0.54 ± 0.02	8.01
1% Ag@PVP	0.69 ± 0.02	15.25 ± 0.25	0.30 ± 0.01	3.2
Ref.	0.71 ± 0.03	12.57 ± 0.20	0.52 ± 0.01	5
1 min	0.65 ± 0.03	14.43 ± 0.1	0.66	6.21
2 min	0.69 ± 0.02	20.73 ± 0.1	0.50	7.14
3 min	0.61 ± 0.03	10.04 ± 0.2	0.64	4.01
0.33% Ag@PVP (2 min)	0.69 ± 0.02	20.73 ± 0.1	0.50 ± 0.02	7.14
0.33% Ag + 0.33% QD (2 min)	0.65 ± 0.01	14.43 ± 0.1	0.66 ± 0.01	6.21
0.33% Ag + 0.33% QD (3 min)	0.71 ± 0.03	21.25 ± 0.2	0.5 ± 0.01	7.65

**Table 2:** The effect of Ag@PVP, and Ag@PVP/CdS QDs on transport, recombination times, and charge-collection efficiency.

DSCs	τ_c(s)_	τ_r (s)_	η_cc_
Reff	1.003E-4	6.02E-4	0.81
0.33 Ag@PVP (2 min)	0.012E-4	0.43E-4	0.97
Ag@PVP/CdS QDs (2 min)	0.825E-4	0.0082E-4	0.99
Ag@PVP/CdS QDs (3 min)	0.0088E-4	0.894E-4	0.99

**Table 3:** Electrochemical parameters obtained by fitting the impedance spectra of DSSCs.

DSCs	τ (s)	K_eff (S_^−1^)	R_CE_(Ω cm^2^)	R_CT_(Ω cm^2^)	Deff (cm^2 ^s^−1^)
CdS QDs	0.075	13.33	45	120	3.55E-5
Reff.	0.041	23.81	14	73	12.7E-5
CdS QDs/Ag@PVP	0.043	23.25	28	110	9.13E-5

## References

[b1] O’Regan B. & Gratzel M. A low-cost, high-efficiency solar cell based on dye-sensitized colloidal TiO_2_ films. Nature 353, 737–740 (1991).

[b2] Zhao G. & Kozuka H., Yoko. Effects of the incorporation of silver and gold nanoparticles on the photoanodic properties of rose bengal sensitized TiO_2_ film electrodes prepared by sol-gel method. Solar Energy Mat. & Sol. Cel. 46, 219–231 (1997).

[b3] Gratzel M. Photoelectrochemical cells. Nature 414, 338–344 (2001). 11713540 10.1038/35104607

[b4] Ismail A. A. & Bahnemann D. W. Mesoporous titania photocatalysts: preparation, characterization and reaction mechanisms. J. Mat. Ch. 21, 11686–11707 (2011).

[b5] Rengifo-Herrera J. A. & Pulgarin C. Photocatalytic activity of N, S co-doped and N-doped commercial anatase TiO_2_ powders towards phenol oxidation and E. coli inactivation under simulated solar light irradiation. Sol. En. 84, 37–43 (2010).

[b6] Jia H., Xu H., Hu Y., Tang Y. & Zhang L. TiO_2_@CdS core–shell nanorods films: Fabrication and dramatically enhanced photoelectrochemical properties. El. Com. 9, 354–360 (2007).

[b7] de Jongh P. E. & Vanmaekelbergh D. Trap-Limited Electronic Transport in Assemblies of Nanometer-Size TiO2 Particles. Phys. Rev. Letts. 77, 3427–3430 (1996). 10062217 10.1103/PhysRevLett.77.3427

[b8] Schlichthörl G., Huang S. Y., Sprague J. & Frank A. J. Band Edge Movement and Recombination Kinetics in Dye-Sensitized Nanocrystalline TiO_2_ Solar Cells: A Study by Intensity Modulated Photovoltage Spectroscopy. J. Phys. Ch. B 101, 8141–8155 (1997).

[b9] Bessho T. *et al.* New Paradigm in Molecular Engineering of Sensitizers for Solar Cell Applications. JACS. 131, 5930–5934 (2009). 10.1021/ja9002684 19334729

[b10] Bomben P. G., Robson K. C. D., Sedach P. A. & Berlinguette C. P. On the Viability of Cyclometalated Ru(II) Complexes for Light-Harvesting Applications in Che. 48, 9631–9643 (2009). 10.1021/ic900653q 19775163

[b11] Johansson P. G. *et al.* Long-Wavelength Sensitization of TiO2 by Ruthenium Diimine Compounds with Low-Lying π* Orbitals. Langmuir. 27, 14522–14531 (2011). 21913708 10.1021/la202887h

[b12] Zhao H. C. *et al.* Evaluation of a Ruthenium Oxyquinolate Architecture for Dye-Sensitized Solar Cells. J. In Ch. 51, 1–3 (2011). 10.1021/ic201375k 22128820

[b13] Gangishetty M. K., Lee K. E., Scott R. W. J. & Kelly T. L. Plasmonic Enhancement of Dye Sensitized Solar Cells in the Red-to-near-Infrared Region using Triangular Core–Shell Ag@SiO_2_ Nanoparticles. ACS Ap. Mat. & Int. 5, 11044–11051 (2013). 10.1021/am403280r 24102234

[b14] Yella A. *et al.* Porphyrin-Sensitized Solar Cells with Cobalt (II/III)–Based Redox Electrolyte Exceed 12 Percent Efficiency. Science. 334, 629–634 (2011). 22053043 10.1126/science.1209688

[b15] Hagfeldt A., Boschloo G., Sun L., Kloo L. & Pettersson H. Dye-Sensitized Solar Cells. Ch. Rev. 110, 6595–6663 (2010). 10.1021/cr900356p 20831177

[b16] Grätzel M. Recent Advances in Sensitized Mesoscopic Solar Cells. Acc. Ch. Res. 42, 1788–1798 (2009). 10.1021/ar900141y 19715294

[b17] Hamann T. W., Jensen R. A., Martinson A. B. F. & Van Ryswyk H Hupp J. T. Advancing beyond current generation dye-sensitized solar cells. En. & Env. Sci. 1, 66–78 (200).

[b18] Barnes W. L., Dereux A. & Ebbesen T. W. Surface plasmon subwavelength optics. Nature 424, 824–830 (2003). 12917696 10.1038/nature01937

[b19] Singh T., Pandya D. K. & Singh R. Surface plasmon driven enhancement in UV-emission of electrochemically grown Zn1−xCdxO nanorods using Au nanoparticles. J. Al. & Comp. 552, 294–298 (2013).

[b20] Derkacs D., Lim S. H., Matheu P., Mar W. & Yu E. T. Excitation of fluorescence decay using a265 nm pulsed light-emitting diode: Evidence for aqueous phenylalanine rotamers. App. Phys. Letts. 89, 655–661 (2006).

[b21] Pillai S., Catchpole K. R., Trupke T. & Green M. A. Surface plasmon enhanced silicon solar cells. J. App. Phys. 101, 101–105 (2007).

[b22] Chen Z. *et al.* Vertically Aligned ZnO Nanorod Arrays Sentisized with Gold Nanoparticles for Schottky Barrier Photovoltaic Cells. The J. of Phys. Ch. C 113, 13433–13437 (2009).

[b23] Catchpole K. R. & Polman A. Plasmonic solar cells. Opt. Exp. 16, 21793–21800 (2008). 10.1364/oe.16.021793 19104612

[b24] Smith W. *et al.* The effect of Ag nanoparticle loading on the photocatalytic activity of TiO2 nanorod arrays. Ch. Phys. Lett. 485, 171–175 (2010).

[b25] Zhang J. & Noguez C. Plasmonic Optical Properties and Applications of Metal Nanostructures. Plasmonics 3, 127–150 (2008).

[b26] Kamat P. V. Quantum Dot Solar Cells. Semiconductor Nanocrystals as Light Harvesters. J. Phys. Ch. C 112, 18737–18753 (2008).

[b27] Hodes G. Comparison of Dye- and Semiconductor-Sensitized Porous Nanocrystalline Liquid Junction Solar Cells. J. Phys. Ch. C 112, 17778–17787 (2008).

[b28] Amiri O., Salavati-Niasari M., Farangi M., Mazaheri M. & Bagheri S. Stable Plasmonic-Improved dye Sensitized Solar Cells by Silver Nanoparticles Between Titanium Dioxide Layers. El. Acta 152, 101–107 (2015).

[b29] Dloczik L. *et al.* Dynamic Response of Dye-Sensitized Nanocrystalline Solar Cells: Characterization by Intensity-Modulated Photocurrent Spectroscopy. J. Phys. Ch. B 101, 10281–10289 (1997).

[b30] Cao F., Oskam G., Meyer G. J. & Searson P. C. Electron transport in porous nanocrystalline TiO_2_ photoelectrochemical cells. J. Phys. Ch. 100, 17021–17027 (1996).

[b31] Schlichthörl G., Park N. G. & Frank A. J. Evaluation of the Charge-Collection Efficiency of Dye-Sensitized Nanocrystalline TiO_2_ Solar Cells. J. Phys. Ch. B 103, 782–791 (1999).

[b32] Amiri O., Salavati-Niasari M., Rafiei Al. & Farangi M. 147% improved efficiency of dye synthesized solar cells by using CdS QDs, Au nanorods and Au nanoparticles. RSC Adv. 4, 62356–62361 (2014).

[b33] Amiri O., Salavati-Niasari M. & Farangi M. Enhancement of Dye-Sensitized solar cells performance by core shell Ag@ organic (organic = 2-nitroaniline, PVA, 4-choloroaniline and PVP): Effects of shell type on photocurrent. El. Acta 153, 90–96 (2015).

